# A high-density linkage map and sex-determination loci in Pacific white shrimp (*Litopenaeus vannamei*)

**DOI:** 10.1186/s12864-024-10431-x

**Published:** 2024-06-05

**Authors:** Baltasar F. Garcia, Vito A. Mastrochirico-Filho, Jousepth Gallardo-Hidalgo, Gabriel R. Campos-Montes, Thania Medrano-Mendoza, Psique Victoria Rivero-Martínez, Alejandra Caballero-Zamora, Diogo T. Hashimoto, José M. Yáñez

**Affiliations:** 1 São Paulo State University (Unesp), Aquaculture Center of UNESP, Jaboticabal, SP 14884-900 Brazil; 2 Facultad de Ciencias Veterinarias y Pecuarias, Universidad de Chile, Santiago, 8820000 Chile; 3 Departamento de El Hombre y su Ambiente, Universidad Autónoma Metropolitana, Unidad Xochimilco, Calzada del Hueso 1100, Coyoacán, CDMX, C.P. 04960 México; 4 Doctorado en Ciencias Agropecuarias, Universidad Autónoma Metropolitana, Unidad Xochimilco, Calzada del Hueso 1100, Coyoacán, CDMX, C.P. 04960 México; 5 Departamento de Producción Agrícola y Animal, Universidad Autónoma Metropolitana, Unidad Xochimilco, Calzada del Hueso 1100, Coyoacán, CDMX, C.P. 04960 México

**Keywords:** Genetic map, GWAS, Sexual dimorphism, *Litopenaeus vannamei*

## Abstract

**Background:**

Expansion of genomic resources for the Pacific white shrimp (*Litopenaeus vannamei*), such as the construction of dense genetic linkage maps, is crucial for the application of genomic tools in order to improve economically relevant traits. Sexual dimorphism exists in Pacific white shrimp, and the mapping of the sex-determination region in this species may help in future reproductive applications. We have constructed male, female, and sex-averaged high-density genetic maps using a 50 K single-nucleotide polymorphism (SNP) array, followed by a genome-wide association study (GWAS) to identify genomic regions associated with sex in white shrimp.

**Results:**

The genetic map yielded 15,256 SNPs assigned to 44 linkage groups (LG). The lengths of the male, female, and sex-averaged maps were 5,741.36, 5,461.20 and 5,525.26 cM, respectively. LG18 was found to be the largest for both sexes, whereas LG44 was the shortest for males and LG31 for females. A sex-determining region was found in LG31 with 21 statistically significant SNPs. The most important SNP was previously identified as a sex-linked marker and was able to identify 99% of the males and 88% of the females. Although other significant markers had a lower ability to determine sex, putative genes were intercepted or close to them. The *oplophorus-luciferin 2-monooxygenase*, *serine/arginine repetitive matrix protein* and *spermine oxidase* genes were identified as candidates with possible participation in important processes of sexual differentiation in shrimp.

**Conclusions:**

Our results provide novel genomic resources for shrimp, including a high-density linkage map and new insights into the sex-determining region in *L. vannamei*, which may be usefulfor future genetics and reproduction applications.

**Supplementary Information:**

The online version contains supplementary material available at 10.1186/s12864-024-10431-x.

## Background

According to the FAO (2023), the Pacific white shrimp (*Litopenaeus vannamei*) holds the distinction of being one of the top aquaculture species in terms of global production. In 2020, approximately 5.81 million tons of this species was cultivated worldwide [1]. White shrimp farming is primarily concentrated in Asia, accounting for 79% of the total production amount. The leading contributor to this continent is China, with a market share of 32%, followed by India (15%) and Indonesia (12%). In the Americas, white shrimp production represents 20% of the global output, with Ecuador having the leading market share, with 13%, followed by Mexico at 3% and Brazil at 1% [1]. The shrimp industry faces several challenges that limit its expansion, such as high incidence of diseases [2], low efficiency of reproduction in captivity [3, 4], adaptation to different rearing environments [5–7], and optimization of production strategies to maximize yield e.g., mono-sex culture [8, 9]. The application of breeding programs and, more recently, genomic tools may help to address these challenges by providing solutions for the shrimp industry, such as production of animals more resistant to diseases, environment-specific genetic lines and production of mono-sex broodstock [10].

An important step towards the implementation of genomic tools in Pacific white shrimp farming is improving the genomic resources of this species. For example, the construction of genetic linkage maps (LM) may help to understand the genetic architecture of important traits [11]. Several LMs have been constructed in regard to the Pacific white shrimp using different types and low numbers of molecular markers. For instance, by using microsatellites, Pérez et al. [12] used 394 amplified fragment length polymorphisms (AFLPs) to construct a preliminary genetic map, and Alcivar-Warren et al. [13] used 100 simple sequence repeats (SSRs) to identify sex-related markers in *L. vannamei*. In addition, an LM using 6,359 specific length amplified fragment sequencing (SLAF) markers was constructed to identify potential quantitative trait loci (QTL) related to body weight [14]. More recently, denser genetic maps have been developed using single nucleotide polymorphism (SNPs with SNP numbers ranging from 418 to 6,146) [7, 15–19]. Most of these previous studies have applied restriction site-associated DNA sequencing (RAD-Seq) and double-digest RAD-Seq. The application of a dense SNP array to genotype animals has not been evaluated in regard to the construction of LM for shrimp.

As in other aquaculture species, the Pacific white shrimp exhibits sexual dimorphism, in which females may have a higher growth rate than males [20]. The genetic mechanism of sex determination in *L. vannamei* and other penaeids was already studied and seems to present a ZZ/ZW model [19, 21]. This model implies that males are homogametic (ZZ), and females are heterogametic (ZW). Genomic studies have been conducted to detect the genomic regions and markers associated with sex determination in the Pacific white shrimp. A sex-determining SNP was first found to be fully associated with linkage group (LG) 18 using linkage mapping analysis of a single family with 205 full-sibs [19]. In a different LG, three other markers were found to be associated with sex in a Mexican commercial population using the RAD-seq method [21, 22]. More recently, Jones et al. [18] found a highly associated SNP that explained more than 79% of the sexual variation. However, the position of this marker was uncertain it was positioned in conjunction with LG42 and 44. Although, some markers have been proposed to be associated with sex determination in this species, there is no consensus regarding the exact position of these markers, and no sex-related genes have been found close to these regions. The objectives of this study were to construct a new LM using a 50 K SNP array, identify genomic regions possibly associated with sex determination through a genome-wide association study (GWAS), and identify potential genes intercepting these regions in the *L. vannamei* genome.

## Methods

### Origin of animals and families

The animals used in this study were provided by the Maricultura del Pacífico hatchery (El Rosário, Sinaloa, Mexico). This breeding population was established in 2013 using animals from different countries (Mexico, Ecuador, Panama, and the USA). This population was selected in order to create two different genetic lines, including white-spot syndrome virus resistance and harvest weight, using pedigree information and estimated breeding values to select animals. A total of eight families (*n* = 1,290) were produced in order to develop this study (mean weight 45.7 g ± 4.98). Animals were identified using elastomers with different color combinations to determine the family origin and were used to generate the LM population with an average number of offspring per family of 20 and a range between 10 and 30. More details on the rearing protocols and production of families can be obtained from Campos-Montes et al. [23] and Castillo-Juárez et al. [24].

### DNA extraction, genotyping and quality control

Pleopods from 1,290 animals were collected and stored in microtubes containing 96% ethanol for 72 h. The samples were sent to the Center of Aquaculture Technologies (CAT) for genomic DNA extraction and genotyping. DNA samples were genotyped using the AquaArray HD (50 K) vannamei® panel developed by Neogen® (Nebraska, USA) resulting in 50,811 SNPs in the raw genotype file.

Quality control (QC) filters were applied to the genotyped SNPs using PLINK 1.9 software [25]. SNPs with a minor allele frequency (MAF) < 0.01 and Hardy-Weinberg equilibrium (HWE) p-value < 1.2E^− 6^ (after Bonferroni correction) were excluded from subsequent analysis. QC filtering also excluded the maximum per-sample (> 0.2) and per-SNP (> 0.1) missing rates. The assignment of the individuals to their respective families was confirmed using CERVUS software [26] and with PLINK to remove SNPs with Mendelian errors (--me 0.1). Samples and markers with high Mendelian errors were removed before map construction.

### Linkage map construction

The modules of the Lep-Map3 software [27] were used in order to construct the LM of *L. vannamei*. *ParentCall2* was used to remove the non-informative SNPs (i.e., SNPs that were homozygous for both parents). *Filtering2* was used to remove the SNPs with significant segregation distortion (data tolerance = 0.001). The SNPs were assigned to 44 linkage groups (LGs) (corresponding to the 44 chromosome pairs in *L. vannamei*) using *SeparateChromosomes2*. In the LG assignment, an optimized LOD score of 20 was achieved using a distortion of LOD = 1. The module *JoinSingles2* was used to assign additional SNPs to the existing LGs, decreasing the LOD scores from 19 to 14 (optimized LOD score = 15). The resulting 44 LGs were ordered using *OrderMarkers2* in order to generate male-, female-, and sex-averaged LMs. LMs were visualized using the LINKAGEMAPVIEW package available in R software [28].

### GWAS for sex determination and gene search

A genome-wide association study (GWAS) for sex determination was performed using the LM coordinates obtained in the previous step for position markers. This population (GWAS population; Table S1) was comprised by 1,049 animals previously subjected to white-spot syndrome virus challenge. Body weight and sex information were available for these animals (44.8% male, and 55.2% female). The DNA extraction and genotyping protocols were the same as those described previously. SNPs that were not assigned to any of the 44 LGs were removed, and the remaining SNPs were repositioned according to the LG and distance (cM) obtained in the LM. Additional QC was applied for genotypes using the following criteria: MAF < 0.05, HWE p-value < 1.2 × 10^− 9^ and maximum rate per-SNP missing < 0.9. The GCTA software [29] was used for the GWAS and a genomic relationship matrix (GRM) was constructed and then included in the model to account for relatedness within the population. The model used was:

$$y = SNPs+e$$

where $y$ is the phenotype (sex, 1 = male and 2 = female), $SNPs$ is the fixed SNP effect, and $e$ is the residual. The *--mlma-loco* (mixed linear model association leaving-one-chromosome-out) function was used because it excludes candidate markers within the same chromosome from the GRM preventing overfitting and increasing statistical power [30]. The P-value of each SNP-association was -log_10_ transformed and visualized using a Manhattan plot using the *qqman* R package [31]. Additionally, linkage disequilibrium (LD) analysis was performed using the top10 SNPs with the lowest p-values to evaluate the effect of historical recombination rates in significant genomic regions within the same LG.

The genotypic frequencies of significant SNPs (lowest p-values) were also evaluated and correlated with sex in order to assess the potential use of these SNPs in determining sex. A nominal logistic model, considering sex as a response variable and genotype as an explanatory variable, was used to estimate the sex detection probabilities for the three most relevant SNPs.

SNP probes of 170 bp corresponding to the top ten significant SNPs, were used to identify any putative genes associated with sex determination. Adjacent probes were BLASTed against the *L. vannamei* reference genome sequence (Zhang et al. [32] - Gen Bank assembly ASM378908v1). Genes found at 50Kb upstream and downstream of the SNP positions were also considered putative genes.

## Results

### QC of genotypes and high-density linkage map

QC of the genotypes was applied separately for the two populations to generate the LM and perform the GWAS (Table 1).

**Table 1:** Quality control of genotypes at individual and SNP level for the high-density linkage map and GWAS populations

Filter (LM population)*	Retained SNPs	Retained Samples
MAF^a^ (> 0.01)	40,141	1,290
HWE^b^ (> 1.2 × 10^− 6^)	40,141	1,290
geno^c^ (< 0.1)	35,801	1,290
mind^d^ (< 0.2)	35,801	1,271
me^e^ (< 0.1)	33,460	748
Filter (GWAS population)^†^		
MAF (> 0.05)	15,034	1,049
HWE (> 1.2 × 10^− 9^)	14,256	1,049
geno (< 0.1)	14,110	1,049
mind (< 0.1)	14,110	1,049

*Initial number of SNPs and samples were 50,811 and 1,290, respectively

^a^ Minor allele frequency

^b^ Hardy-Weinberg equilibrium

^c^ Missing rate at SNP level

^d^ Missing rate at individual level

^e^ Mendelian error rate

^†^ Initial number of SNPs and samples were 15,256 and 1,049, respectively

For the LM population, of the initial 50,811 SNPs, a total of 40,141 SNPs were retained, presenting MAF values higher than 0.01 and on Hardy-Weinberg equilibrium. SNPs exceeding 10% of the missing genotype data were removed, leaving 35,801 SNPs for downstream analyses. Furthermore, 19 individuals exceeded 20% of the missing genotype data and were discarded, resulting in 1,271 individuals for the correct assignment of the true parents. A Mendelian rate test was performed on the individuals to discard alleles that could not be assigned to their putative biological parents. Thus, 2,341 SNPs that were segregated with Mendelian errors in more than 10% of individuals were excluded. Moreover, 523 individuals with more than 10% of SNPs with Mendelian errors were excluded from map construction. After QC filtering step, 33,460 SNPs and 748 individuals from eight families were used to construct the LM.

**Fig. 1:**
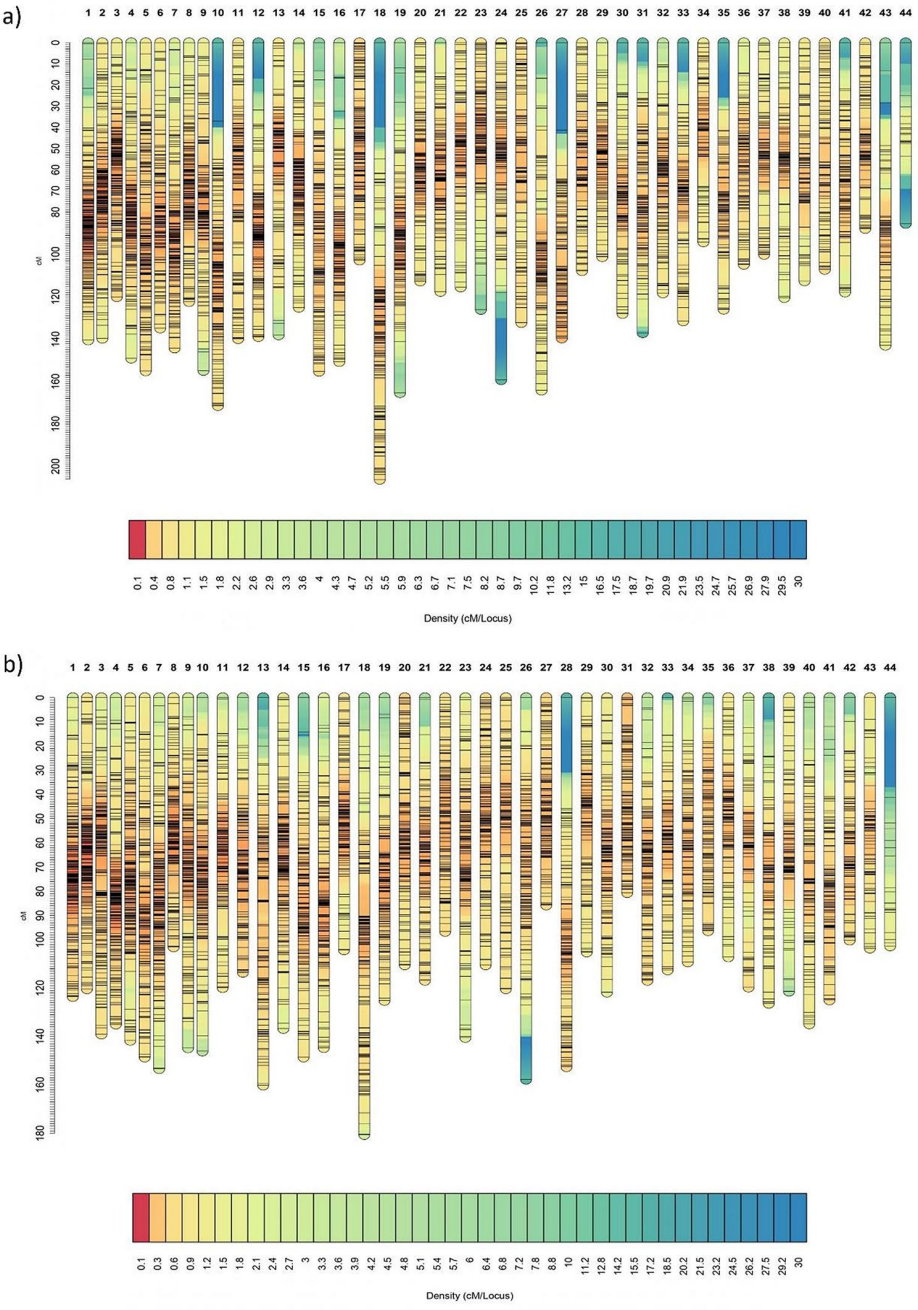
Male (**a**) and female (**b**) linkage maps of *L. vannamei* represented by 44 linkage groups and 15,256 assigned SNPs. Colors represent a density scale of markers ranging from blue (low density regions) to red (high density regions)

For the linkage analysis, the module *ParentCall2* retained a total of 15,289 informative SNPs that were grouped into LGs by the *SeparateChromosomes2* module and expressed at male, female, and sex-averaged levels (Table 2). When an LOD score value of 20 was used, the vast majority of SNPs (15,241) were assigned to 44 LGs (corresponding to 44 pairs of chromosomes in *L. vannamei*), thereafter, a maximum of four markers were assigned to each remaining linkage group. Subsequently, 15 orphan SNPs were assigned to the existing LGs using the *JoinSingles2* module, totalling 15,256 SNPs to be ordered. The marker orders with the highest likelihood for each LG were combined to produce the final linkage maps (Fig. 1a and b). The male, female and sex-averaged maps spanned 5,741.36, 5,461.20 and 5,525.26 cM, respectively, with an average distance between markers of 0.377, 0.359 and 0.365 cM, respectively. LG18 was found to be the largest linkage group for both males (206.22 cM) and females (180.48 cM), whereas LG44 was the shortest for males (85.42 cM) and LG31 was the shortest for females (80.56 cM). The size was determined by measuring the genetic distance between the first and last markers within each LG. The LG that presented the highest density of markers (calculated by subtracting the distance between each marker followed by the overall mean of distances) was LG1 for males (0.242 cM) and females (0.213 cM), and LG44 was the least dense for both sexes, with average marker distance of 3.714 and 4.460 cM, respectively. These metrics indicated that the recombination rate within LG1 was higher than that within LG44, as the presence of more markers imply a higher recombination rate in that specific region.

**Table 2:** Summary of statistics for the high-density male, female and sex-averaged linkage maps of *L. vannamei*

Linkage group (LG)	Number of SNPs	Male		Female		Sex-averaged	
		Length (cM)	Density (cM)	Length (cM)	Density (cM)	Length (cM)	Density (cM)
1	582	140.38	0.242	123.55	0.213	133.54	0.230
2	552	139.62	0.253	120.28	0.218	114.98	0.209
3	491	119.97	0.245	139.05	0.284	123.31	0.252
4	490	149.04	0.305	134.90	0.276	145.43	0.297
5	485	154.96	0.320	141.63	0.293	150.68	0.311
6	468	134.84	0.289	148.48	0.318	127.35	0.273
7	451	144.16	0.320	153.34	0.340	118.05	0.262
8	441	122.27	0.278	102.97	0.234	139.58	0.317
9	439	154.75	0.353	144.69	0.330	161.95	0.370
10	435	171.40	0.395	145.96	0.336	162.95	0.375
11	422	139.84	0.332	119.78	0.284	148.41	0.352
12	413	138.84	0.337	113.48	0.275	110.11	0.267
13	406	138.04	0.341	160.08	0.395	139.61	0.345
14	403	125.04	0.311	136.73	0.340	179.78	0.447
15	399	155.19	0.390	148.51	0.373	134.63	0.338
16	394	150.60	0.383	144.63	0.368	119.03	0.303
17	386	102.65	0.266	104.24	0.271	143.99	0.374
18	378	206.22	0.547	180.48	0.479	146.40	0.388
19	373	165.26	0.444	125.11	0.336	116.14	0.312
20	353	112.57	0.320	110.37	0.314	142.22	0.404
21	346	117.49	0.340	116.84	0.339	132.86	0.385
22	345	115.35	0.335	96.60	0.281	96.10	0.279
23	344	126.00	0.367	140.38	0.409	147.73	0.431
24	341	159.23	0.468	110.42	0.325	104.71	0.308
25	338	132.24	0.392	120.27	0.357	131.76	0.391
26	329	164.05	0.500	157.71	0.481	121.85	0.371
27	319	139.52	0.439	85.71	0.270	106.73	0.336
28	311	107.72	0.347	152.51	0.492	110.07	0.355
29	294	101.05	0.345	105.05	0.358	103.14	0.352
30	294	127.88	0.436	121.68	0.415	120.78	0.412
31	288	137.01	0.479	80.56	0.282	115.91	0.405
32	284	118.21	0.417	116.76	0.412	110.94	0.392
33	264	131.38	0.500	112.52	0.428	114.73	0.436
34	265	94.11	0.356	109.24	0.414	113.20	0.429
35	263	125.90	0.480	96.28	0.367	159.61	0.609
36	258	104.72	0.407	107.12	0.417	111.34	0.433
37	249	99.96	0.403	119.63	0.482	118.84	0.479
38	239	120.44	0.506	126.26	0.530	117.78	0.495
39	229	112.50	0.493	121.31	0.532	132.19	0.580
40	230	107.11	0.467	134.79	0.588	114.34	0.499
41	226	117.75	0.523	124.94	0.555	118.48	0.526
42	218	87.78	0.404	100.25	0.462	89.68	0.413
43	197	142.90	0.729	103.53	0.528	97.19	0.496
44	24	85.42	3.714	102.58	4.460	77.16	3.355

### GWAS and gene search for sex determination

The 15,256 SNPs ordered and positioned in the 44 LGs were extracted from the GWAS population with available sex phenotypes. These SNPs were repositioned according to the sex-averaged LM. A new QC of genotypes was applied, removing 222 SNPs with a MAF lower than 5%, 778 with Hardy-Weinberg disequilibrium, and 46 with a high missing rate (Table 1). A total of 14,110 SNPs and 1,049 samples were available for the GWAS.

**Fig. 2:**
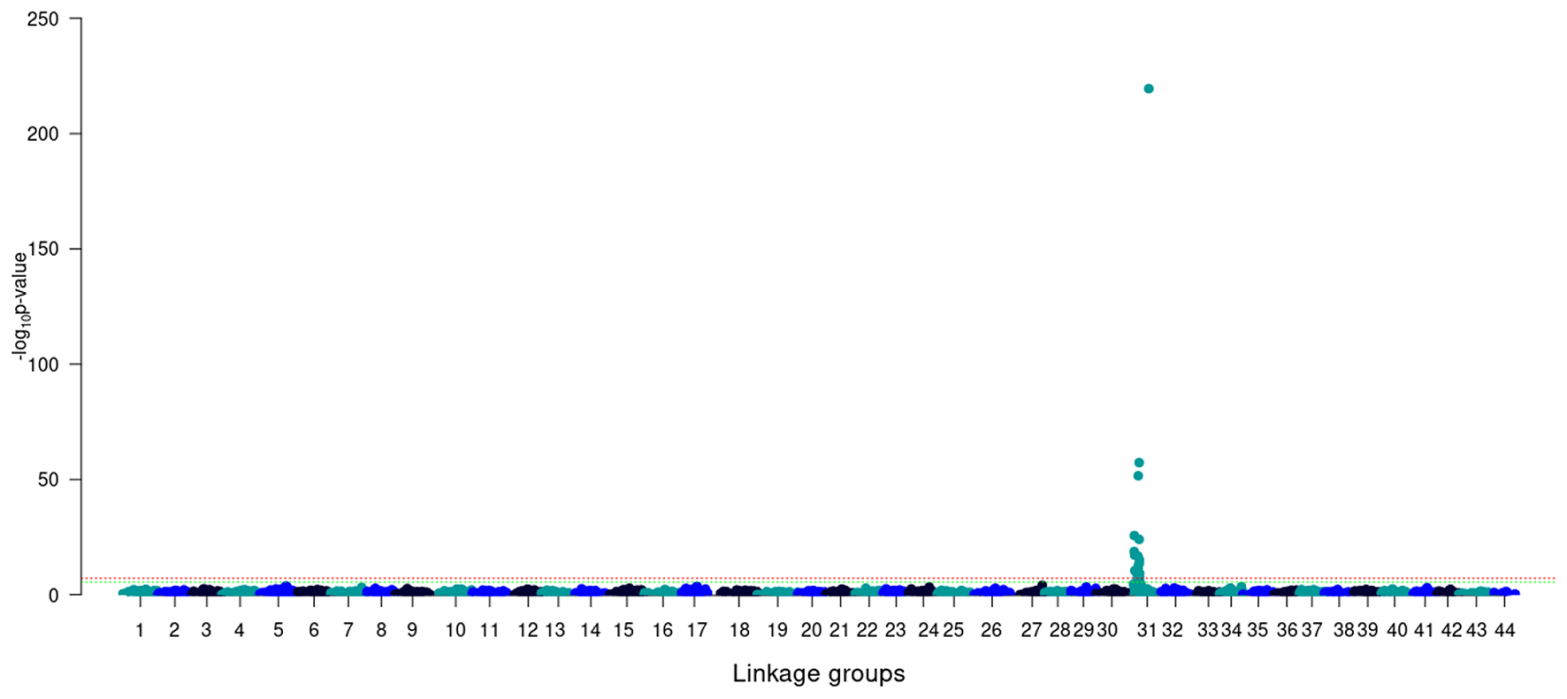
GWAS for sex determination in *L. vannamei* using SNPs positioned through the high-density linkage map (green line: genome-wide significance (0.05/Number of SNPs); red line: chromosomal significance (0.05/Number of SNPs/Number of linkage groups)

The GWAS results showed that LG31 was strongly associated with sex determination in this *L. vannamei* population (Fig. 2). A total of 21 SNPs were found to be significant at the genomic level and the top10 most significant SNPs are listed in Table 3. A complete list of the 21 SNPs is shown in Table S2. The three most significant SNPs showed -log_10_ p-values of 3.3E^− 220^, 5.05E^− 58^ and 2.71E^− 52^, respectively.

**Table 3:** Top-10 SNPs with the lowest p-value associated to sexual determination in *L. vannamei*

LG^a^	Pos (cM)^b^	SNP_ID^c^	*p*-value^d^	Genes^e^
31	86.246	Yu_C19299G_B	3.3E^− 220^	-
31	47.020	AX-249,531,418	5.1E^− 58^	*uncharacterized protein F13E9.13 mitochondrial, COMM domain-containing protein*
31	43.116	AX-249,610,151	2.7E^− 52^	*spermine oxidase*
31	27.065	AX-249,907,556	2.3E^− 26^	*tyrosine-protein kinase CSK, venom protease, acetylcholine receptor*
31	46.700	AX-249,719,810	1.0E^− 24^	-
31	26.457	AX-249,988,993	1.9E^− 19^	**oplophorus-luciferin 2-monooxygenase**
31	28.246	AX-249,687,820	6.8E^− 18^	**serine/arginine repetitive matrix protein**
31	41.544	AX-249,735,384	1.7E^− 17^	-
31	47.491	AX-249,949,377	5.9E^− 16^	-
31	47.376	AX-249,497,067	1.3E^− 14^	*regulator of G-protein signaling loco*

^a^Linkage group

^b^Position in centiMorgans

^c^SNP identification in the 50 K SNP array

^d^P-value of association

^e^Genes intercepted (bold) and close (italic) to significant SNPs within 50Kb up and downstream windows

**Fig. 3:**
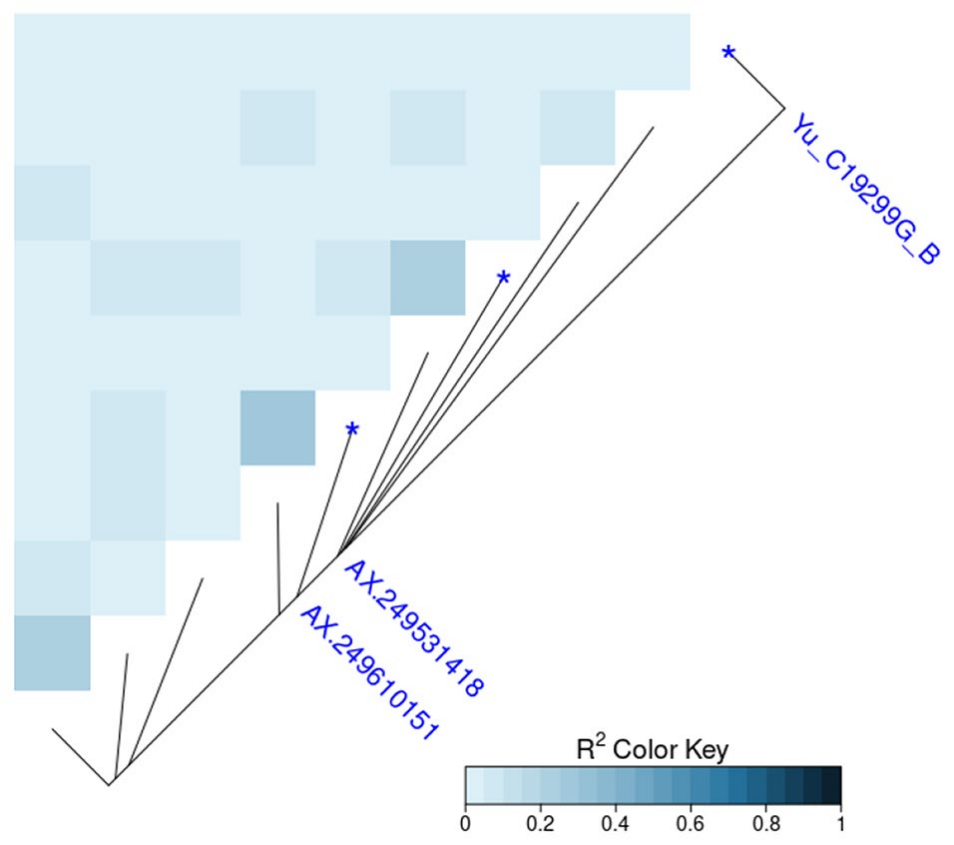
Linkage disequilibrium among the top ten SNPs with the lowest p-values associated to sex determination using the r^2^ metric (in blue the three most significant SNPs)

These SNPs were positioned at 86.246, 47.020, and 43.116 cM LG31, respectively, and, a LD analysis was performed on the top10 significant SNPs to evaluate the level of LD in this genomic region (Fig. 3). A low-to-medium LD level (r² < 0.4) was found among the most relevant SNPs, indicating that they were most likely segregated at different rates.

**Fig. 4:**
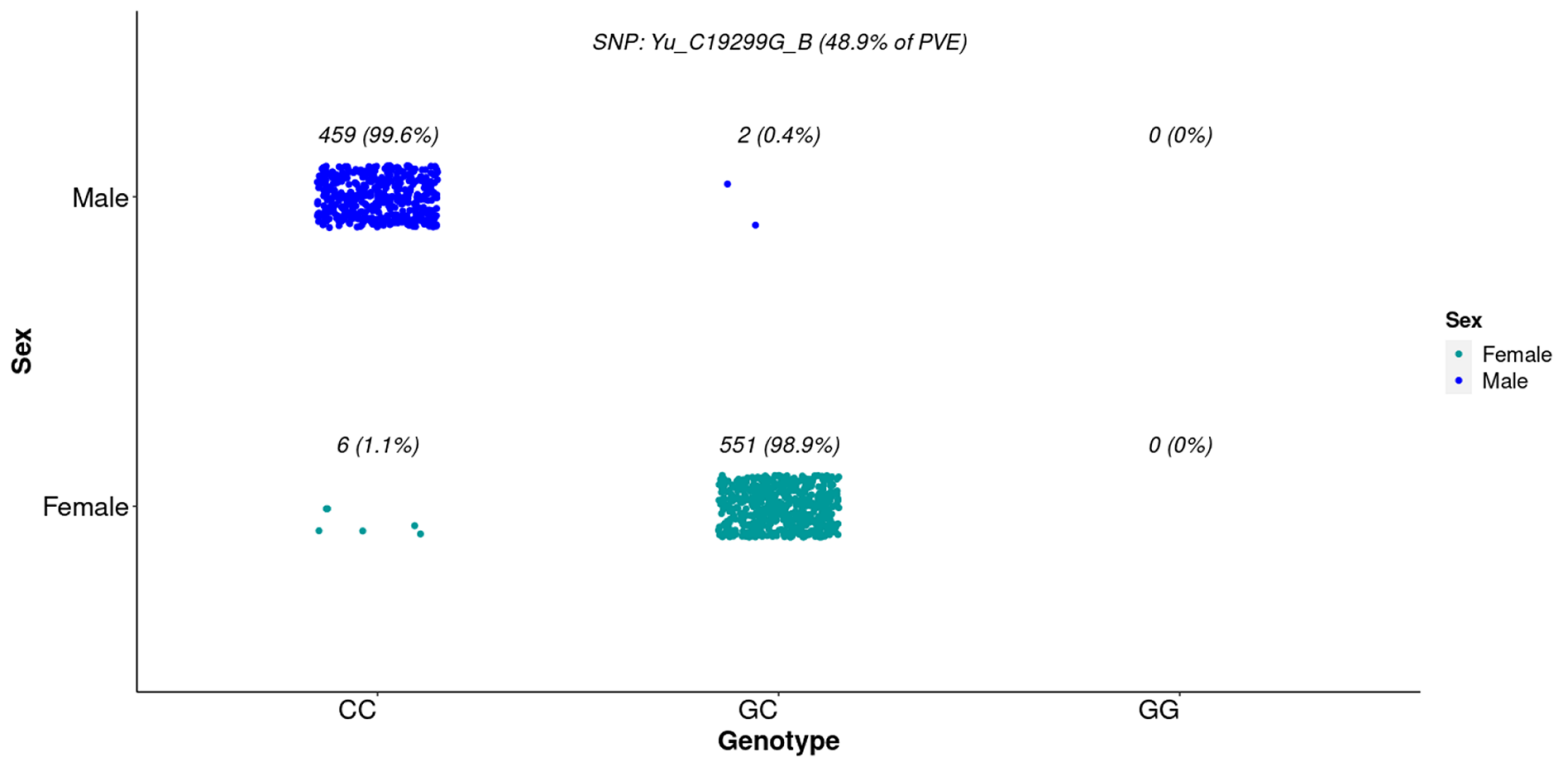
Distribution of phenotypic sex according to the three possible genotypes for the SNP Yu_C19299G_B with the lowest p-value associated to sex in *L. vannamei*

The most significant SNP (Yu_C19299G_B) identified 99% of males (homozygotic) and 88% of females (heterozygotic) (Fig. 4). This marker was already reported as highly associated with sex in a previous study [19]. These results support the proposed ZZ/ZW sex determination system for this species. The other two markers were less reliable in determining sex in the present study, and their potential use in identifing sex could be pruned into errors (Figure S1). Probabilistic analyses revealed a similar trend for sex determination (Table 4).

**Table 4:** Probabilities of sex determination using the three most relevant markers found in the GWAS for *L. vannamei*

Yu_C19299G_B			AX-249,531,418			AX-249,610,151		
Genotype	Male	Female	Genotype	Male	Female	Genotype	Male	Female
CC	0.985	0.015	AA	0.244	0.756	AA	0.229	0.771
CG	0.004	0.996	AT	0.314	0.686	AG	0.340	0.660
GG	-	-	TT	0.977	0.023	GG	0.843	0.157
r²= 0.93			r²= 0.33			r²= 0.20		

Comparing the three most relevant SNPs, Yu’s SNP showed the highest r² (0.93), and the other markers were less reliable in determining sex (0.33 and 0.20, respectively).

The gene search performed using the top ten most significant SNPs intercepted some of the genes annotated in the *L. vannamei* genome. The AX-249,988,993 (LG31, pos: 26.457 cM) and AX-249,687,820 (LG31, pos: 28.246 cM) SNPs intercepted the *oplophorus-luciferin 2-monooxygenase* and *serine/arginine repetitive matrix protein* genes, respectively. Although these genes are not directly related to sex determination, some studies shown that they are expressed in the gonads of crustaceans, such as *Macrobrachium nipponense* [33] and *Penaeus monodon* [34, 35]. Another interesting gene, *spermine oxidase*, was found close to the AX-249,610,151 SNP (~ 20Kb downstream). This gene has also been reported to be differentially expressed in other aquaculture species, including *L. vannamei* [36]. The Yu_C19299G_B SNP was not found close to any gene. However, we used the *blastx* tool with the same scaffold as the input (JANIEY010001236.1) where this SNP was present. Blasting this scaffold against the Zebra fish (*Danio rerio*) and *Daphnia pulex* genomes, the most significant hits (E-value = 2e^− 55^) showed that this region was rich in retrotransposons, transposase, and integrins (Figure S2) such as, reverse transcriptase (RTs), ribonuclease H (RNase H) of the Ty3/Gypsy family, and integrase (H2C2). These elements may be involved in several sexual determination processes as reported in different species.

## Discussion

In the present study, we constructed a high-density linkage map for *L. vannamei* using populations from a breeding program. To the best of our knowledge, this is the densest linkage map constructed for this species using a 50 K SNP array.

Several linkage maps are available for this species, most of which have a relatively low number of SNPs and use RAD-seq methods for genotyping [16–18]. It was proposed that *L. vannamei* presents 44 pseudochromosome pairs [32]. However, some linkage maps showed 45 [11], 48 [17] and even 49 [37] LGs. The present linkage map resulted in 15,256 SNPs distributed in the 44 LGs which is consistent with the genome assembling [32] and previous linkage maps [15, 16]. The LG44 had a significantly lower number of SNPs (24) than to the other LGs (197–587). Penaeids have unique genomic characteristics, including high levels of heterozygosity, polyploidization in some species, and simple sequence repeats accounting for up to 50% of the genome in some cases [38]. Such characteristics, associated with a large genome size (~ 2.6 Gb), have hampered the construction of a high-quality reference genome and the expansion of genome resources for *L. vannamei*.

The male and female linkage maps were found to be very similar in terms of total length and overall mean density. Still, it was possible to observe larger recombination gaps, also known as “coldspots”, in the male when comparing against the female map. This was evident in LG31 (the LG found to be associated with sex determination), where a high recombination rate was observed in the female map, whereas for males, the recombination was more concentrated towards the centromere of the LGs. This pattern is common in many fish species. Cooney et al. [39] evaluated the recombination landscape of 61 fish species and found that 42 species had higher recombination rates in females than males. Although there is no clear adaptative explanation for such differences between sexes, some hypotheses were proposed, such as the meiotic drive (i.e., alleles have an advantage during the process of female meiosis changing recombination rates) and protection against aneuploidy (i.e., failure of chromosomes to separate during cell division) [40–42]. In crustaceans, a previous study showed similar recombination rates between males and females for *Daphnia pulex* [43], which conflicts with our results in shrimp. Thus, there is no “rule of thumb” in the recombination dynamics between sexes. Recombination studies are scarce in crustaceans, especially shrimps, and further investigations are necessary to better understand the recombination landscape of this species.

There is an evident interest in understanding the genetic mechanisms of sexual determination in *L. vannamei*, mainly because of sexual dimorphism in this species. Females have a higher growth rate, and a mono-sex culture may increase the economic return of shrimp farming [20]. The identification of specific genetic regions and genes involved in sex determination may help to manipulate the proportion of females to optimize mono-sex cultures. Associations between markers and sex determination have been reported for *L. vannamei*. Yu et al. [19] identified a genomic region on LG18 related to sexual determination, and a single marker was found to be completely associated with this trait, being a potential SNP for sex characterization in this species. This SNP (Yu_C19299G_B) was present in the 50 K SNP array and it was the most significant SNP identified in this study. Perez-Enriquez et al. [22] also identified three markers associated with this trait using the RAD-seq method to develop a set of SNPs to characterize Mexican shrimp broodstock populations. The three SNPs were evaluated for sex identification of a different population using the post-PCR high-resolution melting method, and a high accuracy of sex determination was reported. Another major effect marker was identified by Jones et al. [18], who reported a strong association with sex; a high percentage of males were homozygotic and females were heterozygous for this marker in a study of almost 2,000 animals. Recently, a new hypothesis was established regarding the sex determination mechanism of *L. vannamei*. The sex-determination regions of males and females of this species were analyzed, and the results indicated that these regions could be influenced by variations in both DNA sequence and genome structure [44]. Using GWAS and the new high-density LM, we identified 21 statistically significant SNPs potentially associated with sex determination in this population at LG31. Yu’s marker was effective in determining of sex in our dataset, confirming the ZZ/ZW determination system and its potential use in other *L. vannamei* populations. These results may help in the fine mapping of the sex determination region, as this important marker was not previously positioned in a high-density genetic map. The other two markers with a higher statistical association with sex determination showed a lower ability to determine sex. A possible explanation for this divergence is the high recombination rate observed in the LM. Most likely, this region experiences strong cross-over events, making it difficult to be detected across multiple families [19]. This can also be visualized in the LD pattern of the most associated SNPs, where low LD was reported among the SNPs in LG31.

In regard to the organisms used in the GWAS, 0.9% (eight females and two males) disagreed with the sex determined in the field (phenotype), with the sex assigned using Yu´s marker. It is possible to infer that these were assignment errors, because when considering AX-249,531,418, sex determination coincided with Yu´s marker. The absence of females with the GC/TT (Yu_C19299G_B / AX-249,531,418) genotype is noteworthy, and could be associated with the presence in this genomic region of genes with an epistatic relationship with lethal effects in females. The main effects of sex-lethal mutations are on the reproductive aspects [45], which were not evaluated in the present study. Because a sex-lethal gene (*Sxl*) has been previously described in this species [46], further investigations of the deleterious effects of these SNPs are necessary.

At least three important genes have been described in the process of sexual determination in *L. vannamei*: sex-lethal (*Sxl*) [46], *Pvfem-1* [47] and *25749_180* loci [21]. Another factor often analyzed for sex determination in shrimp is the level of gene expression according to the developmental phase. For example, Unigene0020898 and Unigene0020336 were found to be differentially expressed at the Zoea I stage, indicating that both genes are likely located in the sex-determining region of *L. vannamei* [48]. *Fem-1* genes were also reported to be important during the Zoea III stage and are possible associated with sex differentiation in females [49]. We did not find any genes close to the top10 significative SNPs identified in the GWAS. However, *oplophorus-luciferin 2-monooxygenase* and *serine/arginine repetitive matrix protein* (SRRM1) genes were intercepted by significant SNPs. The first gene was downregulated at both the PL15 and PL25 stages in the gonadal tissues of *M. nipponense* [33]. Although its specific function is still unclear, this gene was found to be related to metabolic and extra cellular matrix processes in other crustacean species [50, 51]. The second gene was downregulated in the testis and ovary tissues of *P. monodon* using transcriptomic analysis [35]. Serine-arginine proteins have been reported to be differentially expressed during the testis maturation process of the same species [34]. The *spermine oxidase* gene is closely related to a significant SNP. This gene encodes the precursor of spermidine, a well-known polyamine involved in regulating fertility in mammals [52]. The *spermine oxidase* is significantly up-regulated in narrow-clawed crayfish (*Pontastacus leptodactylus*) suggesting its potential role in gonad maturation [53]. It is also differentially expressed in the gonada tissues of *L. vannamei* individuals [36].

The sex-linked SNP identified by Yu et al. [19] and mapped in this study were not associated with any gene. However, a deeper scan of the scaffold in which this SNP was located revealed conserved domains that were possibly associated with sex determination. We found, for instance, a retrotransposon called reverse transcriptase which is a transposable element. Transposable elements are dynamic DNA sequences that play crucial roles in genomic evolution and species diversification [54]. Given their typical expression in the gonads, transposable elements may be particularly prone to participate in sexual development [55]. Another interesting conserved domain found was a Ribonuclease H (RNase H) of the Ty3/Gypsy family. This transposable element may be associated with sex determination and evolution via two approaches: sequence conservation and co-localization with genomic function [56]. The first aspect is related to sex chromosome degeneration. Ty3/Gypsy suggests a potential contribution to the passive process of sex chromosome degeneration, as it was found to accumulate on the W/Y chromosome of other species, possibly starting from nearby insertions in the proximity of crucial heterogametic sex-linked genes [57]. The second aspect is related to co-localization with regions of known genomic functions on sex chromosomes. In fish, SNP association analyses have shown that sex-specific or sex-linked loci are more strongly associated with Ty3/Gypsy than with other transposable elements, as reported in the North African catfish (*Clarias gariepinus*) [58] and bighead catfish (*Clarias macrocephalus*) [59, 60]. In summary, the identification of the Ty3/Gypsy domain in the region where the Yu_C19299G_B SNP is present may indicate a reasonable allignment with the context of the role of these elements in sex chromosome evolution, and thus it supports the hypothesis that Ty3/Gypsy may contribute to structural changes in sex chromosomes.

Our results helped in the identification of genomic regions associated with sex determination in shrimp using informative high-density LM. Future research focused on the fine mapping of this sex-determining region may help disentangle the mechanisms involved in sexual differentiation and the development of mono-sex populations in *L. vannamei*.

## Conclusions

A high-density genetic linkage map for *L. vannamei* species containing 15,256 SNPs, was constructed using a 50 K array. The genetic map allowed the identification of a region highly associated with sex in LG31. A previously reported sex-linked SNP was identified in the same LG with a high accuracy at the sex determination loci in the studied population.

### Electronic supplementary material

Below is the link to the electronic supplementary material.

Supplementary Material 1

Supplementary Material 2

Supplementary Material 3

Supplementary Material 4

## Data Availability

The datasets analyzed during the current study are available in the figshare repository, https://figshare.com/articles/dataset/LM_GWAS_Shrimp/24793677.
