# Supplementary material for: A high-density linkage map and sex-determination loci in Pacific white shrimp (Litopenaeus vannamei)
Source: BMC Genomics. 2024 Jun 5;25:565. doi: 10.1186/s12864-024-10431-x (PMC11155064; doi:10.1186/s12864-024-10431-x)
Supplement: Supplementary file 1 — Supplementary Material 1 [file 12864_2024_10431_MOESM1_ESM.docx]

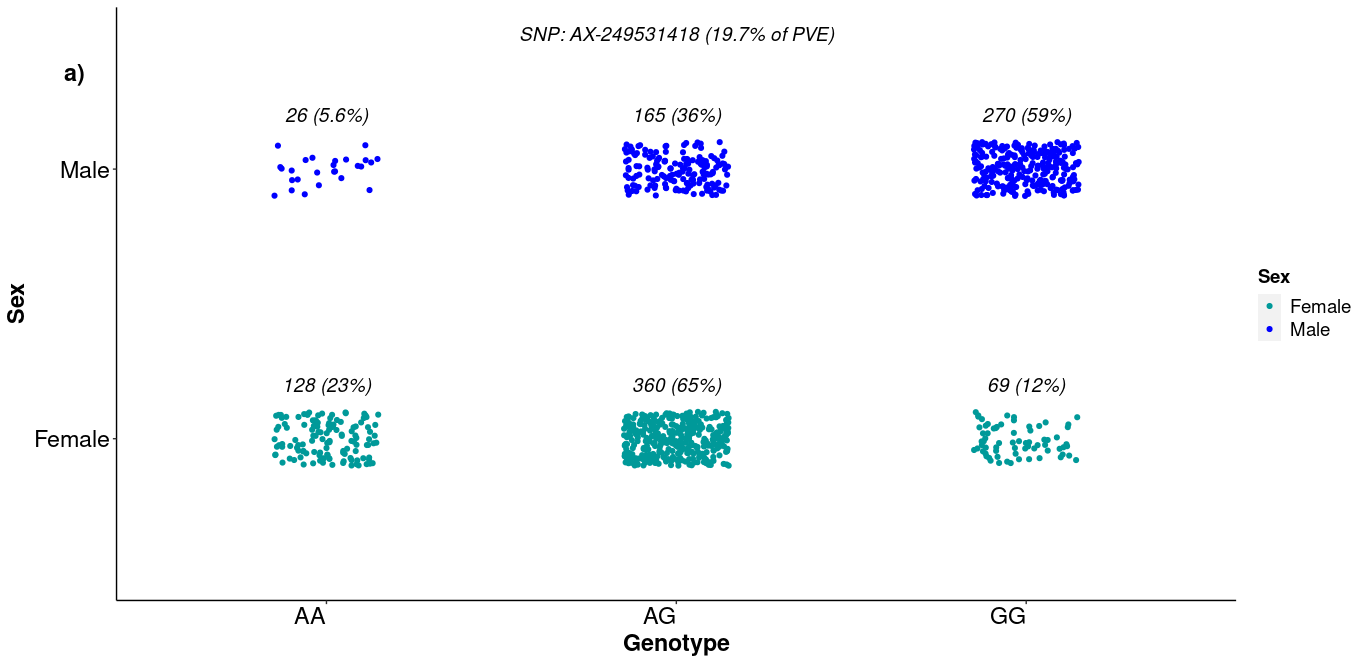

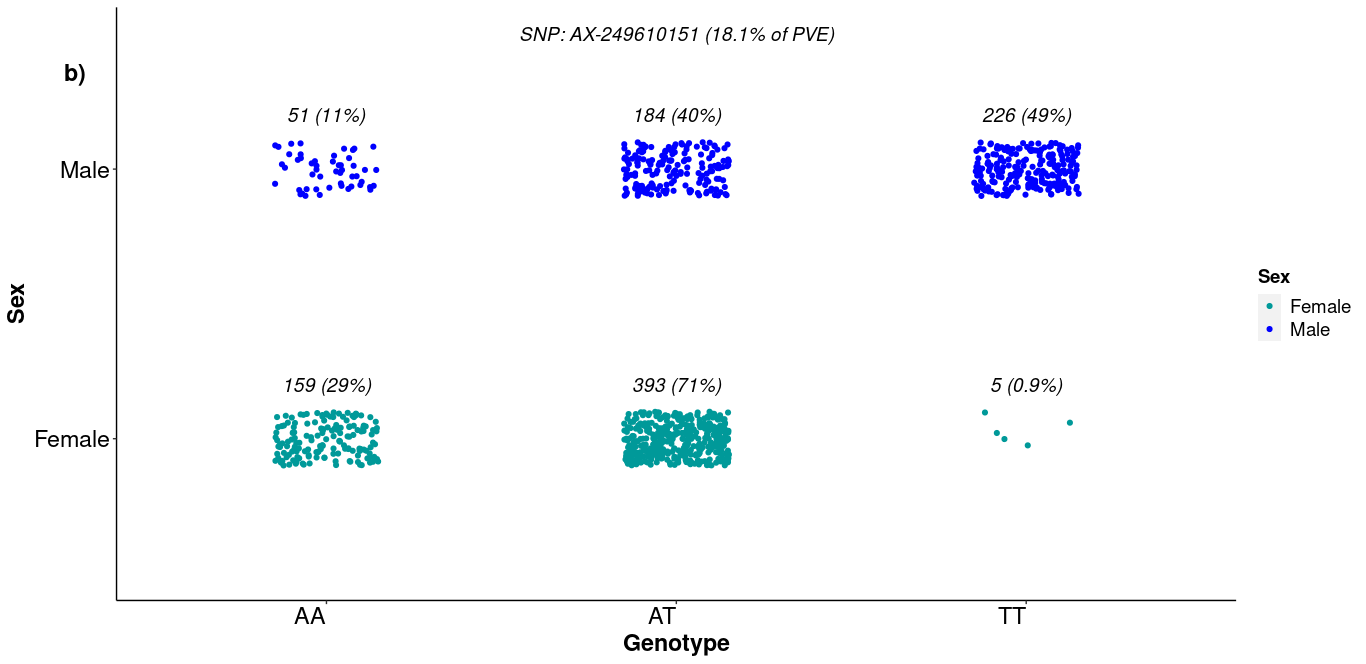


Figure S1. Distribution of *L. vannamei* males and females according to the three possible genotypes for the two SNPs with highest phenotypic variance explained (PVE) (percentages were calculated according to the number of males/females).
