# Supplementary material for: A high-density linkage map and sex-determination loci in Pacific white shrimp (Litopenaeus vannamei)
Source: BMC Genomics. 2024 Jun 5;25:565. doi: 10.1186/s12864-024-10431-x (PMC11155064; doi:10.1186/s12864-024-10431-x)
Supplement: Supplementary file 2 — Supplementary Material 2 [file 12864_2024_10431_MOESM2_ESM.docx]

Table S1. Total number of animals, families, males, females, mean weight (SD) in grams and mean age (SD) in days of animals included in the GWAS analysis (GWAS population).

| Total of animals | 1,049 |
| --- | --- |
| Families | 167 |
| Males | 470 |
| Females | 579 |
| Weight (SD) | 16.2 (2.9) |
| Age (SD) | 127.3 (1.9) |
