# Supplementary material for: A high-density linkage map and sex-determination loci in Pacific white shrimp (Litopenaeus vannamei)
Source: BMC Genomics. 2024 Jun 5;25:565. doi: 10.1186/s12864-024-10431-x (PMC11155064; doi:10.1186/s12864-024-10431-x)
Supplement: Supplementary file 3 — Supplementary Material 3 [file 12864_2024_10431_MOESM3_ESM.docx]

Table S2. Significant SNPs at genomic level for sex determination in *L. vannamei* revealed by GWAS.

| LG^a^ | SNP^b^ | cM^c^ | A1^d^ | A2^e^ | Freq^f^ | b^g^ | b(se)^h^ | p-value^i^ | PVE (%)^j^ |
| --- | --- | --- | --- | --- | --- | --- | --- | --- | --- |
| 31 | Yu_C19299G_B | 86.246 | G | C | 0.27 | 0.98 | 0.03 | 3.40E-220 | 48.89 |
| 31 | AX-249531418 | 47.020 | A | T | 0.49 | 0.38 | 0.02 | 5.06E-58 | 19.73 |
| 31 | AX-249610151 | 43.116 | A | G | 0.41 | 0.35 | 0.02 | 2.71E-52 | 18.08 |
| 31 | AX-249907556 | 27.065 | G | A | 0.28 | 0.26 | 0.02 | 2.32E-26 | 9.71 |
| 31 | AX-249988993 | 26.457 | A | G | 0.45 | 0.21 | 0.02 | 1.92E-19 | 7.19 |
| 31 | AX-249949377 | 47.491 | A | C | 0.39 | 0.18 | 0.02 | 5.92E-16 | 5.87 |
| 31 | AX-249757380 | 42.273 | A | T | 0.29 | 0.17 | 0.02 | 4.02E-12 | 4.39 |
| 31 | AX-249677759 | 29.270 | G | C | 0.48 | 0.15 | 0.02 | 3.66E-11 | 4.01 |
| 31 | AX-249829322 | 45.610 | A | G | 0.39 | -0.13 | 0.02 | 6.86E-08 | 2.70 |
| 31 | AX-249745818 | 46.562 | A | T | 0.50 | -0.13 | 0.02 | 5.14E-09 | 3.15 |
| 31 | AX-249800768 | 48.124 | A | G | 0.29 | -0.14 | 0.02 | 1.89E-08 | 2.92 |
| 31 | AX-249544365 | 42.574 | A | G | 0.32 | -0.14 | 0.02 | 7.97E-09 | 3.08 |
| 31 | AX-250023089 | 43.337 | A | G | 0.23 | -0.14 | 0.03 | 8.10E-08 | 2.67 |
| 31 | AX-249879019 | 45.291 | T | A | 0.49 | -0.17 | 0.02 | 7.52E-14 | 5.06 |
| 31 | 6475_120 | 39.715 | G | A | 0.19 | -0.17 | 0.03 | 3.80E-09 | 3.20 |
| 31 | AX-249464258 | 42.150 | T | A | 0.26 | -0.18 | 0.03 | 1.58E-12 | 4.55 |
| 31 | AX-249981318 | 48.677 | G | A | 0.17 | -0.18 | 0.03 | 5.58E-10 | 3.54 |
| 31 | AX-249735384 | 41.544 | T | A | 0.40 | -0.20 | 0.02 | 1.73E-17 | 6.46 |
| 31 | AX-249497067 | 47.376 | A | C | 0.23 | -0.21 | 0.03 | 1.33E-14 | 5.35 |
| 31 | AX-249687820 | 28.246 | A | C | 0.30 | -0.21 | 0.02 | 6.82E-18 | 6.61 |
| 31 | AX-249719810 | 46.700 | G | A | 0.33 | -0.24 | 0.02 | 1.01E-24 | 9.13 |

^a^ Linkage group.

^b^ SNP identification.

^c^ Position in centiMorgans.

^d^ Allele 1.

^e^ Allele 2.

^f^ Frequency of major allele.

^g^ SNP effect.

^h^ Standard error of SNP effect.

^i^ P-value of association.

^j^ Percentage of phenotypic variance explained.
