# Supplementary material for: A high-density linkage map and sex-determination loci in Pacific white shrimp (Litopenaeus vannamei)
Source: BMC Genomics. 2024 Jun 5;25:565. doi: 10.1186/s12864-024-10431-x (PMC11155064; doi:10.1186/s12864-024-10431-x)
Supplement: Supplementary file 4 — Supplementary Material 4 [file 12864_2024_10431_MOESM4_ESM.docx]

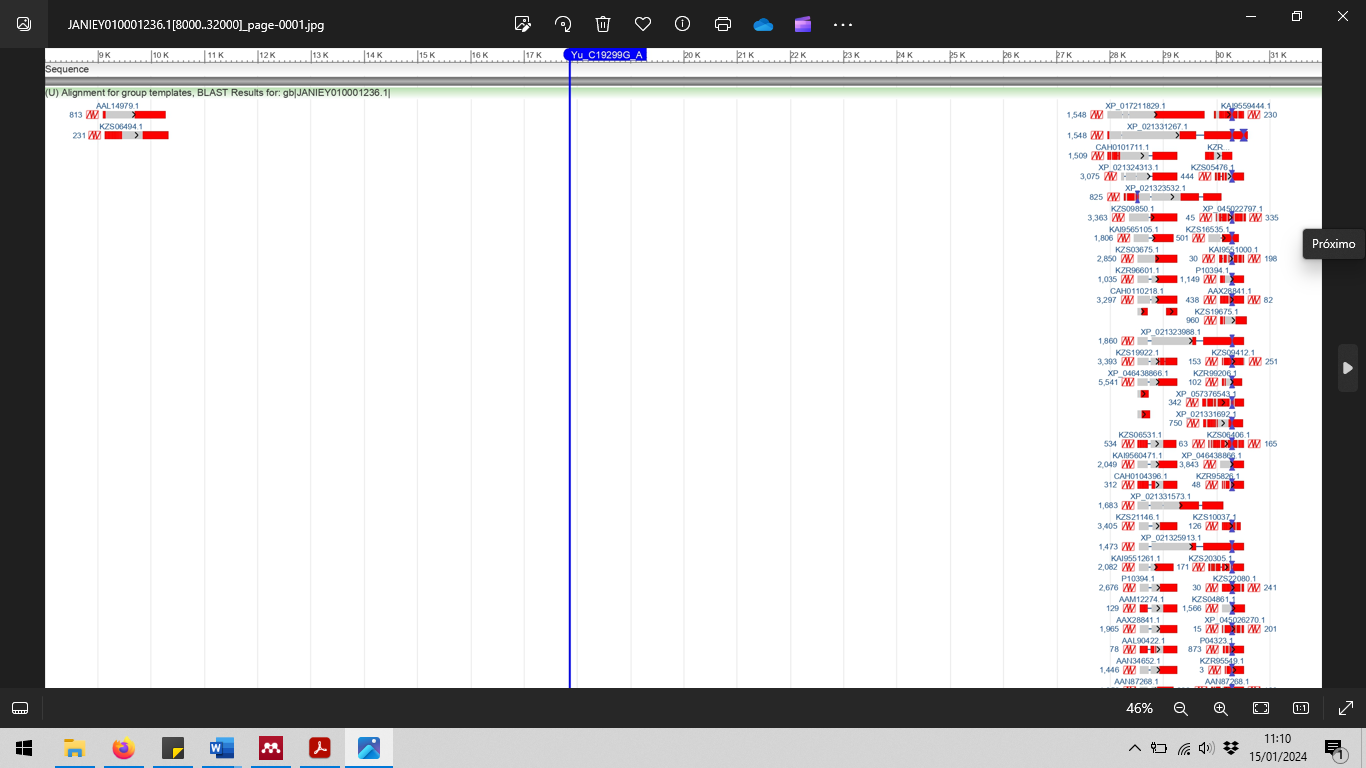


Figure S2. Position of the Yu_C19299G_B SNP and close conserved domains found in the JANIEY010001236.1 scaffold of *L. vannamei*.
